# Examining the Interplay between the Cognitive and Emotional Aspects of Gender Differences in Spatial Processing

**DOI:** 10.3390/jintelligence12030030

**Published:** 2024-03-04

**Authors:** Cynthia M. Fioriti, Raeanne N. Martell, Richard J. Daker, Eleanor P. Malone, H. Moriah Sokolowski, Adam E. Green, Susan C. Levine, Erin A. Maloney, Gerardo Ramirez, Ian M. Lyons

**Affiliations:** 1 Department of Psychology, Georgetown University, Washington, DC 20057, USA; rjd107@georgetown.edu (R.J.D.); ellie.malone@nih.gov (E.P.M.); aeg58@georgetown.edu (A.E.G.); iml30@georgetown.edu (I.M.L.); 2 Department of Psychology, Toronto Metropolitan University, Toronto, ON M5B 2K3, Canada; hm.sokolowski@torontomu.ca; 3 Rotman Research Institute, Baycrest Hospital, North York, ON M6A 2E1, Canada; 4 Department of Psychology, University of Chicago, Chicago, IL 60637, USA; s-levine@uchicago.edu; 5 School of Psychology, University of Ottawa, Ottawa, ON K1N 6N5, Canada; erin.maloney@uottawa.ca; 6 Department of Educational Psychology, Ball State University, Muncie, IN 47306, USA; gramirez6@bsu.edu

**Keywords:** spatial skills, spatial anxiety, gender differences

## Abstract

Women reliably perform worse than men on measures of spatial ability, particularly those involving mental rotation. At the same time, females also report higher levels of spatial anxiety than males. What remains unclear, however, is whether and in what ways gender differences in these cognitive and affective aspects of spatial processing may be interrelated. Here, we tested for robust gender differences across six different datasets in spatial ability and spatial anxiety (N = 1257, 830 females). Further, we tested for bidirectional mediation effects. We identified indirect relations between gender and spatial skills through spatial anxiety, as well as between gender and spatial anxiety through spatial skills. In the gender → spatial anxiety → spatial ability direction, spatial anxiety explained an average of 22.4% of gender differences in spatial ability. In the gender → spatial ability → spatial anxiety direction, spatial ability explained an average of 25.9% of gender differences in spatial anxiety. Broadly, these results support a strong relation between cognitive and affective factors when explaining gender differences in the spatial domain. However, the nature of this relation may be more complex than has been assumed in previous literature. On a practical level, the results of this study caution the development of interventions to address gender differences in spatial processing which focus primarily on either spatial anxiety or spatial ability until such further research can be conducted. Our results also speak to the need for future longitudinal work to determine the precise mechanisms linking cognitive and affective factors in spatial processing.

## 1. Introduction

There is a substantial body of work indicating that males reliably outperform females on certain measures of spatial ability (Hyde 1981; Linn and Petersen 1985; McGee 1979; Uttal et al. 2013; Voyer et al. 1995). This difference in spatial processing is particularly noticeable in mental rotation tasks (MRTs) (Shepard and Metzler 1971) in which participants must mentally rotate two- or three-dimensional objects in space (Lauer et al. 2019; Linn and Petersen 1985; Voyer et al. 1995). In recent years, researchers have also taken a burgeoning interest in the complex interplay between cognitive and affective processes in the spatial domain. Early work in this area has shown that females report higher levels of anxiety about tasks or situations involving spatial processing, or spatial anxiety (Lyons et al. 2018), relative to males (Lauer et al. 2018; Ramirez et al. 2012; Sokolowski et al. 2019). What remains largely unclear, however, is whether and in what ways gender differences in cognitive and affective aspects of spatial processing may be interrelated.

Unpacking the interplay between cognitive and affective factors in spatial processing may be of broader interest for several reasons. First, women continue to be underrepresented in the majority of STEM fields (Beede et al. 2011; Blickenstaff 2005). At the same time, spatial skills have emerged as a stable predictor of successful engagement with science, technology, engineering, and math (STEM) fields (Gunderson et al. 2012; Lubinski 2010; Uttal and Cohen 2012; Wai et al. 2009). Further, spatial anxiety is a unique predictor of poorer spatial skills, especially skills that tend to show the greatest gender differences, such as mental rotation (Daker et al. 2022b). Thus, understanding the interplay between cognition and emotion in the spatial domain may have implications for understanding and addressing the underrepresentation of women in STEM fields.

Very few studies to date have directly investigated how the relation between cognition and emotion in the spatial domain relates to gender differences (McGlone and Aronson 2006; Heil et al. 2012; Neuburger et al. 2015). To our knowledge, however, only one study has examined the intersection between spatial anxiety and spatial performance (Alvarez-Vargas et al. 2020; see Sokolowski et al. 2019 for a related analysis in the context of mathematical processing). In their study, Alvarez-Vargas et al. (2020) conducted a mediation analysis in which they found that spatial anxiety explained (mediated) a significant portion of gender differences in MRT performance. Research in other cognitive domains, such as mathematics, has suggested that a heightened state of anxiety can temporarily disrupt working memory resources, which can lead to reduced performance on tasks in that domain (Ashcraft and Kirk 2001; Dowker et al. 2016; Daker et al. 2023b). Hence, one possible implication of the Alvarez-Vargas et al. (2020) results is that women on average experience greater levels of anxiety when faced with spatial tasks which temporarily disrupt available cognitive resources. In this way, affective factors could help to explain lower average performance on spatial tasks for women relative to men.

An alternative—albeit not mutually exclusive—possibility is that gender differences in anxiety within a given domain are not the cause but the result of poor performance in that domain. For instance, in the math domain, some have suggested that math anxiety is a response to repeatedly experiencing performance-related failures in mathematics (Gunderson et al. 2018; Maloney and Beilock 2012; Núñez-Peña and Suárez-Pellicioni 2014; Ramirez et al. 2018). Consistent with this claim, reciprocal relations have been observed in primary school children wherein lower math performance at Time 1 predicts higher math anxiety at Time 2, controlling for math anxiety at Time 1 (Gunderson et al. 2018; Song et al. 2021). In the spatial domain, it may similarly be the case that increased reports of spatial anxiety in women is a response to an awareness among women that, on average, they experience poorer performance-related outcomes when engaging in spatial tasks and activities. In other words, it may be the case that gender differences in spatial ability explain (mediate) gender differences in spatial anxiety—i.e., the reverse of what Alvarez-Vargas et al. (2020) showed. To our knowledge, no prior work has directly tested this hypothesis.

### Current Study

The goals of the current study were two-fold. First, given the limited research explicitly exploring connections between gender differences, spatial ability, and spatial anxiety, we aimed to test two hypotheses: (1) that gender differences in spatial anxiety explain (mediate) gender differences in spatial ability; (2) that gender differences in spatial ability explain (mediate) gender differences in spatial anxiety. Second, we sought to establish robust estimates of each potential mediation effect by testing both hypotheses across a range of datasets involving a diverse set of populations. Namely, we examined six separate cross-sectional datasets involving a grand total of 1257 participants. We report the mediation effects of individual datasets, the average of the mediation effects across all six datasets, and the average effect sizes of gender on spatial anxiety and ability. While this study does not investigate causality, it helps to lay the groundwork for future causal investigations. Findings from the current study provide the most comprehensive test to date of the interrelation between gender differences in cognitive and affective aspects of spatial processing.

## 2. Methods

### 2.1. Participants

Participant recruitment procedures differed across studies. Table 1 lists the analytic sample size, gender ratio, and subject age for the examinations in this manuscript. Participants in each study were asked to report their self-identified gender association rather than their sex. We, therefore, use the term gender throughout this manuscript. Our final analytic N across all studies is 1257 (830 females), with individual studies ranging in number of participants from 105 to 385. The mean age across all studies is 19.42 years.

### 2.2. Procedure

Each study obtained metrics of participant mental rotation ability and spatial anxiety. General anxiety was also collected as a control measure. Background information including participant age and self-reported gender was collected. In all cases, these metrics were part of larger datasets. The order of presentation for surveys and cognitive tasks was counterbalanced in each study. Five of the six datasets have been used previously in published works (Table 1; Cortes et al. 2022; Daker et al. 2022b; Lyons et al. 2018; Daker et al. 2022a). All analyses presented here are novel and address hypotheses specific to this current study.

#### 2.2.1. GSS Dataset

The GSS dataset was obtained via a classroom intervention created in partnership between public high schools in Virginia and the Integrated Science and Technology Department at James Madison University. The GSS collects data at two timepoints: pre-curriculum (T1) and post-curriculum (T2). Our manuscript utilizes the data collected from the control group, which are those students who did not engage with the GSS curriculum, during the post-curriculum follow-up session (T2). We do this because measures of MRTs, spatial anxiety, and trait anxiety were not collected from the majority of participants at T1 but were at T2. Tasks in this study were counterbalanced between timepoints, between participants, and within a single session. All data were collected in person, and spatial and general anxiety surveys were administered on paper to the students. The MRTs were administered via computer. Details on study procedures for MRTs in the GSS Study can be found in the Supplementary Materials of Cortes et al. (2022). The GSS Study procedures were approved by the Georgetown University Institutional Review Board (IRB).

#### 2.2.2. Western Dataset

The Western dataset consists of a sample of first-year undergraduate students at the University of Western Ontario in London, Canada. Data were collected in person, with spatial and general anxiety responses collected via a computerized survey, and MRTs were collected via a computerized task. Details regarding study procedures for both the survey and MRTs can be found in Daker et al. (2022b). Study procedures were approved by the Western University Ethics Review Board.

#### 2.2.3. Ottawa Dataset

The Ottawa dataset consists of a sample of undergraduate students enrolled in a psychology course at the University of Ottawa. Participants received research credit for their participation. Survey data on spatial and general anxiety were collected via computer in the lab. MRTs were also administered in person via computer. More detailed information on experimental procedures can be found in Daker et al. (2022b). Study procedures were approved by the University of Ottawa Office of Research Ethics.

#### 2.2.4. UCLA Dataset

The ULCA dataset consists of a sample of students at the University of California, Los Angeles. Survey data on spatial and general anxiety were collected via computer in the lab. MRTs were also administered in person via computer. More detailed information on the experimental procedures can be found in Lyons et al. (2018). The UCLA Study was approved by the UCLA IRB.

#### 2.2.5. Georgetown Dataset

The Georgetown dataset consists of Georgetown University undergraduate students recruited via Georgetown University’s online participant recruitment database. Participation was entirely online through Qualtrics. Survey data on spatial and general anxiety, and MRT performance data were collected via the Qualtrics platform. As this dataset is yet unpublished, see the Measures Section below for further details. Study procedures were approved by the Georgetown University IRB.

#### 2.2.6. GU-CS Dataset

The GU-CS dataset consists of a sample of undergraduate and graduate students enrolled in introductory and advanced computer science courses at Georgetown University. Participants completed an online battery to collect both spatial and general anxiety measures and MRT performance. More detailed information on the experimental procedures for this study can be found in Daker et al. (2022a). Study procedures were approved by the Georgetown University IRB.

### 2.3. Measures

#### 2.3.1. Mental Rotation Task (MRT)

In all 6 studies, spatial ability was measured using a computerized version of the mental rotation task. In 5 of the datasets (GSS, Western, Ottawa, Georgetown, GU-CS), spatial ability was measured using the Shepard and Metzler (1971) version, in which participants view two line drawings of abstract three-dimensional figures made up of cubes on a computer screen. Participants were asked to indicate whether the figures were the same figure, just rotated in space, or were different figures. Studies differed slightly in the ratio of true (same figure) to false (different figure) trials that were used. Objects were rotated along one of the x, y, and z axes, and the degree to which objects were rotated varied across trials. In these five studies (GSS, Western, Ottawa, Georgetown, GU-CS), the score for MRTs is the participant accuracy rate for the task where a higher score indicates better performance.

The sixth dataset (UCLA) used the Weisberg et al. (2014) version of the MRT task, in which participants viewed one line drawing of an abstract three-dimensional figure made up of cubes on the left side of a screen and four probe figures on the right side of the screen. Two of the probe figures were the same as the figure on the left, just rotated in space; two were foils (different figures). Participants needed to determine which 2 probes were the same as the left-most figure, just having been rotated. Participants received feedback if they chose the incorrect probe. Objects were rotated along one of the x, y, and z axes, and the degree to which objects were rotated varied across trials. For this study, hit-rates (H) and false-alarm-rates (FA) were computed across all trials for each participant. H is the proportion of items where a person correctly indicated the figures were the same, and FA is the proportion of items where a person incorrectly indicated the figures were the same. These rates were then used to compute d-prime (or “sensitivity”) estimates via the formula d’ = Z(H) − Z(FA), where Z(x) corresponds to the inverse of the cumulative (Gaussian) distribution function (Stanislaw and Todorov 1999). A higher value of d’ is indicative of better MRT performance.

#### 2.3.2. Spatial Anxiety

All six studies used the Spatial Anxiety Scale (SAS; Lyons et al. 2018). This scale consists of 24 items which measure anxiety about three subtypes of spatial reasoning: mental manipulation, spatial navigation, and spatial imagery (8 items each). Here we focus on just the mental manipulation subscale, as items on this scale correspond most closely with the type of processing needed for our measure of spatial ability–MRT performance. For simplicity, hereafter we refer to ratings on this scale simply as ‘spatial anxiety’, though it may be useful to keep in mind that we are referring specifically to anxiety about situations requiring spatial mental manipulation. A higher rating on this measure indicates higher anxiety (range: 0–32).

#### 2.3.3. General Anxiety

All six studies used the Trait subscale of the State-Trait Anxiety Inventory (STAI; Spielberger et al. 1970) to measure general anxiety. This subscale consists of 20 items and participants are asked to indicate how much each statement applies to them. A higher rating on this scale indicates higher general anxiety (range: 20–80). Both general anxiety and spatial anxiety are typically higher among women (Knowles and Olatunji 2020). Therefore, controlling for general anxiety helps analytic models to distinguish the unique effect of spatial anxiety from the effect of general anxiety and minimizes confounds (Daker et al. 2021).

#### 2.3.4. Age

All six studies collected age as a continuous variable. Age is included as a covariate in our mediation models as research has shown age-related differences in the magnitude of gender differences in mental rotation ability (Levine et al. 2016; Voyer et al. 1995) such that gender-related differences in spatial ability are stronger in adults (18+ years) than in adolescents (13–18 years).

### 2.4. Analysis Framework

#### 2.4.1. Mediation Analysis

To examine the consistency of evidence for each of our two hypotheses, we tested each hypothesis separately in each of our separate datasets (see below for details concerning multiple comparisons). We first assess whether a relation exists between the primary variables of each mediation direction, testing for the presence of gender effects in both spatial anxiety and spatial ability.

Once such relations have been established, we compute the mediation effects for each study, for each hypothesis, in R-studio using Andrew Hayes’ PROCESS macro (Hayes [2013] 2022). PROCESS’s bootstrapping approach utilizes a randomized number simulator when generating results. All mediation pathway effects were run using the PROCESS bootstrapping method (with 5000 iterations) to generate standard errors and confidence intervals. To generate reproducible results, we set our random seed for all mediation models at 404,516. By default, PROCESS generates robust estimates using bootstrapping for the indirect effect in a mediation model. Here, we also generate bootstrapped confidence intervals for all other paths in our models. We control for the effect of general anxiety and age when testing both hypotheses by including this variable as a covariate in all mediation models. Covariates are included in the estimation of all model pathways (a, b, c, c’, and thus also ab).

Only participants with complete datasets for present purposes (gender identification, age, MRT, spatial anxiety, and general anxiety scores) were considered. Note also that the PROCESS macro does not automatically generate standardized total (c) and direct (c’) pathway coefficients when the IV variable is binary (which it was in our case: gender). Hence, these coefficients were computed separately using standardized input variables via regression models that correspond to those used for the total and direct paths in the mediation framework. Validity checks indicated that other model components (e.g., A and B paths) were equivalent when comparing manual regression and PROCESS outputs.

Here it is important to emphasize that the use of mediation in the present context is not meant to provide implicit or explicit evidence for causality. Rather, mediation refers to the extent to which a given third variable is statistically responsible for reducing the observed association between two other variables. To put it another way, imagine running a regression model and observing a particular association between an IV and the DV. One then adds a new IV to the model and observes that the relation between the first IV and the DV has changed. One might ask, what is the precise magnitude of that change, and is it statistically robust? A mediation analysis in the form of the indirect effect answers these questions. In the present context, we are effectively asking (1) to what extent does adding spatial anxiety to a model with gender as the IV and spatial ability as the DV reduce the relation between gender and spatial ability; and (2) to what extent does adding spatial ability to a model with gender as the IV and spatial anxiety as the DV reduce the relation between gender and spatial ability? Quite crucially, as these are cross-sectional mediation analyses, we do not propose to interpret the outcomes of any of our models as being causal or that the strength of a given mediation direction insinuates causality or the “correctness” of a mediation direction. Furthermore, here we use terms like ‘unidirectional’ and ‘bidirectional’ *not* to imply causal direction, but instead to delineate asymmetrical vs. symmetrical mediation effects.

#### 2.4.2. Multiple Comparisons

With respect to multiple comparisons, it is useful to note the probability of observing a given number of significant (p < 0.05) results. For a given hypothesis, we conducted 6 separate tests of that hypothesis. The probability of observing a single significant result supporting that hypothesis by chance alone is p = 0.265. The probabilities of observing 2–6 significant results for that hypothesis by chance alone are p = 0.033, p = 0.002, p = 8.6 × 10^−5^, p = 1.8 × 10^6^, and p = 1.6 × 10^−8^, respectively. Here, we are primarily concerned with the overall pattern of results. Thus, we consider 2 significant results as weak evidence in favor of a given hypothesis (p = 0.033); 3 significant results as moderate evidence in favor of a given hypothesis (p = 0.002), and 4 or more results as strong evidence in favor of a given hypothesis (p ≤ 8.6 × 10^−5^)^1^.

#### 2.4.3. Data Standardization

To allow for direct comparison of mediation effects (as well as the constituent pathways) across datasets, we first standardized all continuous input measures (MRT, spatial anxiety, general anxiety). We did so for each measure in each dataset separately, using the usual normalization procedure: $z_{i}=\frac{x_{i}-M}{s}$, where *M* and *s* are the sample mean and standard deviation, respectively. Note that the standardization procedure included only participants with complete datasets—i.e., only those included in the analysis.

#### 2.4.4. Gender Coding

Surveyed participants were asked to report their gender as either “male”, “female”, or “non-binary.”^2^ In the Georgetown University Online Study, one participant endorsed the “non-binary” selection. Given that our analyses aim to assess the gender dynamics of spatial cognition, we chose to exclude this participant from our analyses. We arbitrarily chose to code our gender variable so that “female” = 1 and “male” = 0. This means that a positive path between gender and another variable indicates scores on that variable were higher for those identifying as female; a negative path indicates the opposite; a path that is not significantly different from 0 indicates no gender difference for the measure in question.

## 3. Results

The goal of this study is to examine gender differences in the relation between cognitive and affective elements of spatial cognition. To begin this examination, we first explored all six datasets to determine whether there existed (1) gender differences in spatial ability, and (2) gender differences in spatial anxiety. Regarding mediation, these gender effects establish whether there is a total effect (C-Path) between X and Y, or predictor and outcome, in both our mediation directions. Consequently, this same examination establishes the presence of the a-path in each mediation model. Finally, in each dataset, we tested the extent to which the presence of spatial anxiety in the model reduces (mediates) the relation between gender and spatial ability; and conversely, the extent to which the presence of spatial ability in the model reduces (mediates) the relation between gender and spatial anxiety.

### 3.1. Establishing Gender Effects

#### 3.1.1. Gender Differences in Spatial Ability

**Figure 1:**
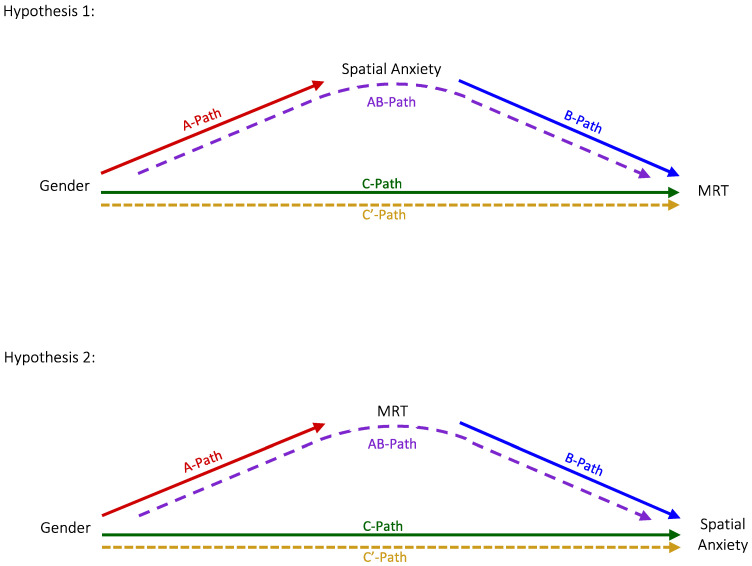
Figure provides a systematic illustration of the mediation models for each hypothesis. Hypothesis 1 is that gender differences in spatial anxiety explain (mediate) gender differences in spatial ability. Hypothesis 2 is that gender differences in spatial ability explain (mediate) gender differences in spatial anxiety.

Hypothesis 1 is that gender differences in spatial anxiety mediate gender difference in spatial ability (see Figure 1). Here we examined the presence of gender differences in spatial ability, which is also the total effect (C-Path) in the mediation model for Hypothesis 1.

**Figure 2:**
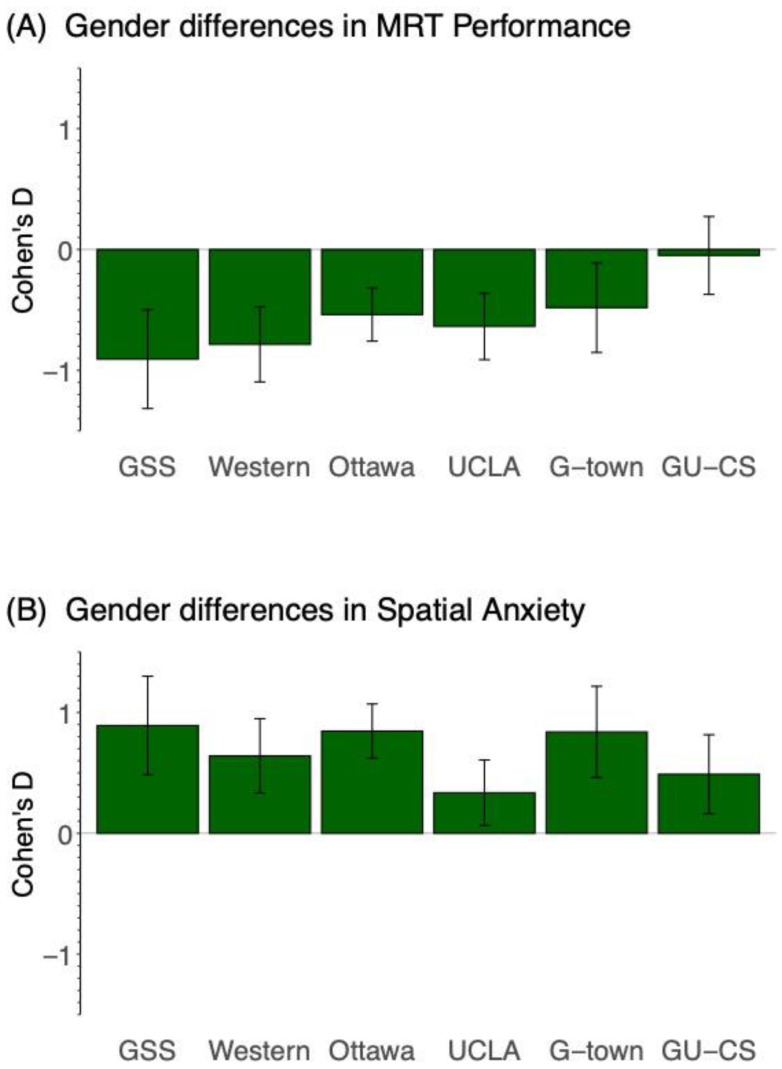
Figure shows the effect size (Cohen’s d) of the gender difference in spatial ability (**A**) with women consistently having lower MRT performance than men, and in spatial anxiety (**B**) with women consistently having higher spatial anxiety than men. A positive value indicates higher spatial anxiety for women than men; a negative bar indicates the reverse. The GU-CS dataset deviates from this, where there are no gender differences in spatial ability.

Figure 2 shows the effect sizes (Cohen’s d) for gender differences in spatial ability in each of the six datasets. A positive value indicates higher spatial performance or anxiety for women than men; a negative bar indicates the reverse. See Appendix A, Table A1 for full statistical details. In five of the six datasets, we found that women have significantly lower MRT performance than men. The lone exception was the GU-CS dataset, where we did not find a significant gender difference in MRT performance (p = 0.763, d = −0.049). The average gender effect size, weighted by sample size, across the remaining five datasets was −0.56, with a range from −0.987 to −0.353. The probability of observing five of six significant effects by chance is p = 1.8 × 10^−6^, therefore we consider this strong evidence for lower MRT performance for women relative to men. This result is aligned with past work which identifies robust gender effects on spatial ability. We identify a gender effect in spatial ability in five of our six datasets, establishing the presence of the total effect (C-Path) for Hypothesis 1 in these datasets. There was no total effect (C-Path) of gender for MRT performance in the GU-CS dataset.

#### 3.1.2. Gender Differences in Spatial Anxiety

Hypothesis 2 is that gender differences in spatial ability mediate gender difference in spatial anxiety (see Figure 1). Here we establish the presence of gender differences in spatial anxiety, which is also the total effect (C-Path) in the mediation model for Hypothesis 2.

Figure 2 shows the effect sizes (Cohen’s d) for gender differences in spatial anxiety in each of the six datasets. A positive value indicates higher spatial anxiety for women than men; a negative bar indicates the reverse. See Appendix A, Table A2 for statistical details. In all six datasets, we found that women report significantly higher spatial anxiety than men. The average gender effect size, weighted by sample size, across all six datasets was 0.67, with a range from 0.354 to 0.993. The probability of observing six of six significant effects by chance is p = 1.6 × 10^−8^, so we consider this strong evidence for higher spatial anxiety among women relative to men. To our knowledge, this is the first use of multiple comparisons to identify the strength of gender effects on spatial anxiety. These results identify the presence of the total effect (C-Path) for Hypothesis 2 in all six datasets.

#### 3.1.3. Spatial Anxiety and Spatial Ability

We tested the correlation between spatial anxiety and spatial ability to evaluate the relation between the outcome variable and the mediator of both mediation directions (see correlations of all variables and covariates in Appendix B, Table A3). In five of the six datasets, spatial anxiety was significantly negatively correlated with spatial ability (range of r was −0.333 to −0.291), such that higher spatial anxiety was associated with lower spatial ability. In the GU-CS dataset, spatial anxiety was not correlated with spatial ability (−0.113, p = 0.169).

## 4. Mediation Results

Having established the pattern of gender effects and the relation between spatial anxiety and ability in our six datasets, we proceeded by testing our mediation hypotheses in all six datasets. Note that, as with the results of gender effect sizes (Cohen’s d), we also report the average of our mediation pathway coefficients across datasets for each mediation direction (Table 2 and Table 3). Importantly, these effects must not be statistically compared, as doing so may lead to erroneous conclusions regarding the “correctness” of a given mediation direction (Lemmer and Gollwitzer 2017).

**Hypothesis** **1:** *Do gender differences in spatial anxiety explain (mediate) gender differences in spatial ability?*

**Figure 3:**
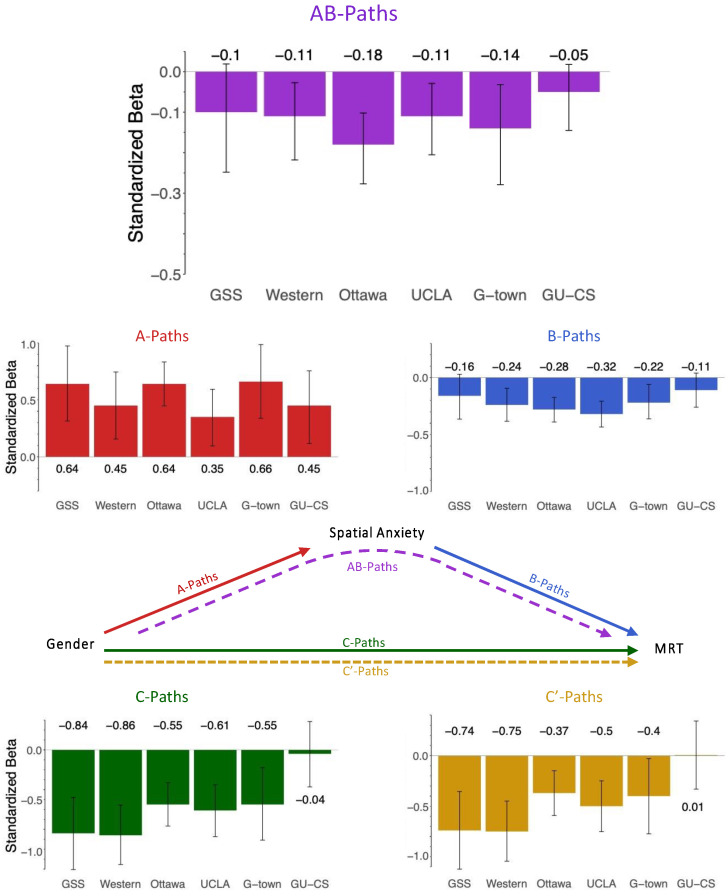
Figure shows the mediation pathway coefficients for all 6 datasets. The indirect effects (AB-Paths) for each dataset are shown in purple. Error bars are 95% confidence intervals. In 4 of the 6 datasets shown here, spatial anxiety significantly mediates the relation between gender and MRT performance. The indirect effect is not significant in 2 of the 6 datasets: GSS and GU-CS. Percent mediation in each dataset can be obtained by dividing the indirect effect (AB-Paths) by the total effect (C-Path) and are shown in Appendix C, Table A4.

Figure 3 depicts the results of the mediation model for Hypothesis 1, and Table 2 reports the average mediation effects. Mediation results for individual datasets are in Appendix C. All reported models control for age and the effect of trait anxiety. The pattern of mediation results remains the same both with and without the covariate of trait anxiety.

For Hypothesis 1, we found a significant indirect effect in four of six datasets. Those datasets without significant indirect effects are the GU-CS dataset and the GSS dataset. Table 2 contains the averages of each mediation path (unweighted and weighted by sample size), both including and excluding those datasets without evidence of mediation (GU-CS and GSS). In the text, we report the weighted average pathway coefficients for all six datasets.

In these models, the average gender difference in spatial anxiety (A-Path) was 0.531 (range: 0.353 to 0.657), and the average effect of spatial anxiety on spatial ability (B-Path) was −0.244 (range: −0.161 to −0.318). The a-path was significant for all datasets, and the b-path was significant for four of the six datasets tested, except the GSS and GU-CS datasets. Gender had a significant total effect (C-Path) on spatial ability in five of the six datasets, except GU-CS, with an average effect of −0.571 (range: −0.044 to −0.855). Spatial anxiety explained (mediated) a significant portion of the link between gender and spatial ability (AB-Path) in four of the six datasets with an average indirect effect of −0.128 (range: −0.05 to −0.178). Spatial anxiety accounted for an average of 22.4% of the effect between gender and spatial ability. After introducing spatial anxiety as a mediator, the residual direct effect (C’-Path) of gender on spatial ability remained significant in five datasets, except the GU-CS dataset which did not originally have a significant total effect, with an average direct effect of −0.443 (range: −0.006 to −0.746).

**Hypothesis** **2:** *Do gender differences in spatial ability explain (mediate) gender differences in spatial anxiety?*

**Figure 4:**
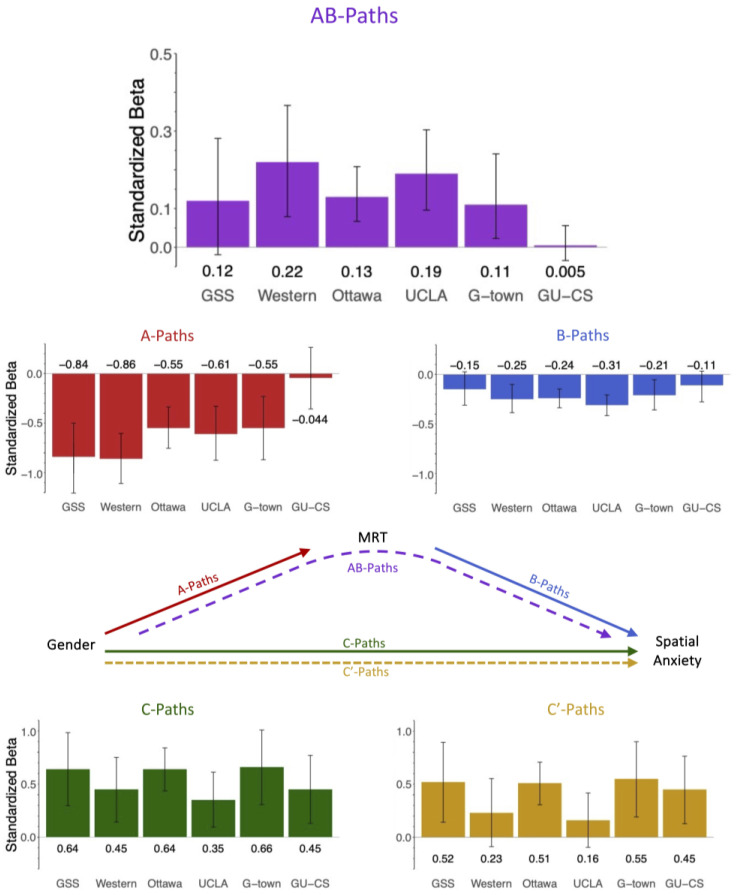
Figure shows the mediation pathway coefficients for all 6 datasets. The indirect effects (AB-Paths) for each dataset are shown in purple. Error bars are 95% confidence intervals. In 4 of the 6 datasets shown here, MRT performance (spatial ability) significantly mediates the relation between gender and spatial anxiety. The indirect effect is not significant in 2 of the 6 datasets: GSS and GU-CS. Percent mediation in each dataset can be obtained by dividing the indirect effect (AB-Paths) by the total effect (C-Path) and are shown in Appendix D, Table A5.

Figure 4 depicts the results of the mediation model for Hypothesis 2 and Table 3 reports the average mediation effects. Mediation results for individual datasets are in Appendix D. All reported models control for age and the effect of trait anxiety. The pattern of mediation results remains the same both with and without the covariate of trait anxiety.

For Hypothesis 2, we found a significant indirect effect in four of six datasets. Those datasets without significant indirect effects are the GU-CS dataset and the GSS dataset. Table 3 contains the averages of each mediation path (unweighted and weighted by sample size), both including and excluding those datasets without evidence of mediation (GU-CS and GSS). In the text, we report the weighted average pathway coefficients for all six datasets.

In our mediation model, the average gender difference in spatial ability (A-Path) was −0.571 (range: −0.044 to −0.855), and the average effect of MRT performance on spatial anxiety (B-Path) was −0.227 (range: −0.105 to −0.313). The a-path was significant in five of the six datasets, excluding GU-CS. The b-path was significant in four of the six datasets, excluding GSS and GU-CS. Gender had a significant total effect (C-Path) on spatial anxiety in all datasets, with an average effect of 0.531 (range: 0.353 to 0.657). Spatial ability explained (mediated) a significant portion of the link between gender and spatial anxiety (AB-Paths) in all four datasets with an average indirect effect of 0.138 (range: 0.005 to 0.215). Spatial ability accounted for an average of 25.9% of the effect between gender and spatial anxiety. After introducing spatial ability as a mediator, the residual direct effect (C’-Path) of gender on spatial anxiety remained significant in four of the six datasets with an average direct effect of 0.394 (range: 0.161 to 0.546).

### Hypothesis 1 and Hypothesis 2

In Hypothesis 1, gender differences in spatial anxiety explain an average of 22.4% of gender differences in spatial ability. In Hypothesis 2, gender differences in spatial ability explain an average of 25.9% of gender differences in spatial anxiety (see Table 2 and Table 3). These are the averages across all six datasets weighted by sample size. The relative magnitudes of the model directions remain intact when comparing both the weighted and unweighted averages of all six datasets and just the four datasets with significant indirect effects.

Readers must note that average mediation effects should be used in a conceptually similar way to effect sizes (for example, Figure 1), providing readers a magnitude by which to interpret the average mediation effect identified within a mediation direction. Average mediation effects must not be statistically compared. The resistance of average mediation effects to statistical comparison directly results from the resistance of singular indirect effects to statistical comparison. The strength and significance of the indirect effects between each mediation direction for a given dataset (and thus, for average mediation effects of a direction across datasets) is not an indicator of the “correctness” or causality between factors of a given direction (Lemmer and Gollwitzer 2017).

Average mediation effects should not be statistically compared between directions as this can lead to erroneous conclusions about causality. Rather, their magnitude may be interpreted within a given mediation direction, providing readers with a single value by which to summarize the detected mediation effects within a given direction. To our knowledge, only one mediation direction has been tested in the current literature (gender → spatial anxiety → spatial ability). This suggests an implicit bias toward a specific causal model in the literature. Our results provide just as much evidence for the reverse direction (gender → spatial ability → spatial anxiety), evidencing that such a bias is unwarranted. Present evidence, both here and in the extant literature cannot distinguish between the “correctness” of these two mediation directions. Rather, the similarity in magnitude of the average mediation effects serves to caution against premature causal interpretations of the relation between gender, spatial anxiety, and spatial ability.

## 5. Discussion

Current literature supports the conclusion that females perform worse on measures of spatial ability, particularly mental rotation (Linn and Petersen 1985; McGee 1979; Uttal et al. 2013; Voyer et al. 1995) and report higher levels of spatial anxiety (Lauer et al. 2018; Ramirez et al. 2012; Sokolowski et al. 2019) than their male counterparts. However, to our knowledge, only one study has investigated the potential relation between these gender differences. In that study, Alvarez-Vargas et al. (2020) showed that gender differences in spatial anxiety explained a significant portion of (i.e., mediated) gender differences in spatial ability. This suggests that, with respect to spatial processing, gender differences in the affective domain can help explain gender differences in the cognitive domain. However, gender differences can be contentious, so it is especially important to accumulate substantial evidence before making strong claims about the nature of gender-related effects.

Here we sought to fill the aforementioned gap in two ways. First, we tested for the mediation effect reported by Alvarez-Vargas et al. (2020) across six separate and unique datasets (total *N* = 1257). Second, we tested for the reverse mediation: that gender differences in the cognitive domain can explain gender differences in the affective domain (also across the same six datasets). To our knowledge, no study to date has investigated this latter possibility. As such, the current study provided a robust test of the following two hypotheses: (1) gender differences in spatial anxiety mediate gender differences in spatial ability; (2) gender differences in spatial ability mediate gender differences in spatial anxiety. Consistent with Alvarez-Vargas et al. (2020), our results provided robust support for the first hypothesis. Results also provided as much evidence in favor of the second hypothesis. These results underscore two points. First, cognitive and affective factors appear to be strongly intertwined when it comes to gender differences in spatial processing. Second, our results urge caution in drawing strong conclusions about the direction of influence between cognitive and affective factors in this respect. The most plausible interpretation of our data is that these influences may be bidirectional given that we cannot use our cross-sectional data to rule out one direction or the other. This suggests that future longitudinal and intervention work is needed to draw specific conclusions about directionality.

The results of this study indicate that when it comes to gender differences in spatial processing, there is a strong relationship between cognitive and affective factors. This is consistent with current trends in the literature which point to reliable interactions between cognitive and affective processing at both the behavioral and neural levels (Dolcos et al. 2011; Lerner et al. 2015; Ochsner and Gross 2005; Phelps 2006; Storbeck and Clore 2007). With respect to gender differences in the spatial domain, accounting for both cognitive and affective differences may be particularly useful in identifying factors underlying disparities in avoidance of activities a person believes involve spatial thinking, such as various STEM fields (Daker et al. 2023a). Against this backdrop, research has shown that spatial skills are important for successful engagement with STEM fields (Gunderson et al. 2012; Lubinski 2010; Uttal and Cohen 2012). Research has also shown that women tend to be underrepresented in STEM fields (Beede et al. 2011; Blickenstaff 2005). Hence, some researchers have proposed that gender differences in spatial processing can help explain disparities in STEM representation (Halpern et al. 2007; Reilly et al. 2017). In order to more fully understand how spatial processing may contribute to this disparity, our results indicate that considering both its cognitive and affective aspects may be key.

### 5.1. Explaining the Exceptions

Despite gender differences in spatial processing being robust, they are not universal. We showed evidence of this when only five of our six datasets supported gender differences in spatial ability, the exception being the GU-CS dataset. Note that the participants in this dataset were all students actively enrolled in computer science coursework at Georgetown University at the time of their participation. Our results suggest that we can infer, in non-specialized groups, that there are consistent gender effects in spatial ability, with women demonstrating worse performance than men. However, in specialized subgroups, such as the individuals in the GU-CS sample, there may be no gender differences in spatial ability. With respect to the GU-CS sample, the lack of this effect may be due to the spatial cognition required for successful engagement with computational coursework. We cannot assert that spatial coursework such as computer science classes mitigates gender differences in spatial ability that would otherwise exist. We are equally unable to assert that women who enroll in computer science coursework have higher levels of spatial ability than the broader population of women.

For the five datasets that did show significant gender effects, four of these showed significant bidirectional mediation effects. The exception was the GSS dataset, which showed significant gender effects (C-Paths) but nonsignificant indirect effects (AB-Paths). One notable point about the GSS dataset is that participants were high school students, with an average age of 16.61 years. This stands in contrast to all other datasets whose samples were made entirely of university students and adults over the age of 18. One possibility is that participant age and developmental stage may influence the relationships between gender, spatial ability, and spatial anxiety (for results consistent with this view, see Levine et al. 2016, Wai et al. 2009, and Voyer et al. 1995). However, more specific work investigating these relations across age groups and developmental stages would need to be conducted to better understand these results.

### 5.2. A Note on Causation

For those populations in which gender effects are evident, in addition to investigating both cognitive and affective aspects of spatial processing, our results indicate that it is important to consider multiple ways in which these factors may be related. Current literature on gender differences in spatial processing has tended to focus primarily on a unidirectional relation in which affective processing influences cognitive processing (Alvarez-Vargas et al. 2020; Lauer et al. 2018; Ramirez et al. 2012). Concomitantly, a variety of potential intervention suggestions that aim to improve spatial ability by targeting spatial anxiety have emerged (Lauer et al. 2018; Ramirez et al. 2012). While our results are not inconsistent with this view, providing robust evidence that gender differences in spatial anxiety mediate gender differences in mental rotation performance, we argue that the clearest interpretation of our bidirectional effects is that inferences about causality are at best premature. That is, our data are also consistent with an alternative framework by which low spatial ability exerts an upward influence on spatial anxiety. Crucially, we are not saying that our cross-sectional results are clear evidence for one causal model or the other (indeed, non-intervention-based data of any kind are unable to do so). Instead, perhaps another way of looking at our results is what we did or did not find evidence against.

To that aforementioned end, consider that while it is possible to have correlation without causation, it is far less likely to have the reverse (i.e., causation without correlation). While we provided evidence that gender differences in spatial anxiety mediate gender differences in mental rotation performance, we also showed the reverse (i.e., that gender differences in mental rotation ability mediate gender differences in spatial anxiety). In other words, our results showed evidence of mediation effects in both directions (affective → cognitive and cognitive → affective). Had we failed to show a mediation effect in one direction, we may have cast doubt on the existence of a potential causal relation in that direction given that there cannot be causation without a correlative counterpart. However, in showing evidence of mediation effects in both directions, it would seem imprudent to rule out either causal direction. This is relevant for the current literature because an overemphasis on one causal direction or another (as noted above) may be seen as implicitly assuming that alternative causal relations (and hence alternative forms of intervention) are implausible. Our results demonstrate it would be imprudent to do so. At the risk of overstepping, we would suggest that, given the current state of the literature barring future evidence to the contrary, perhaps the most prudent path forward would be to systematically explore interventions targeting both cognitive and affective components (separately and together).

A precedent for the importance of considering such a potential bidirectional relation exists in the math cognition literature. Research on math cognition has long identified a relation between math anxiety/attitudes and math ability (Ashcraft and Krause 2007; Devine et al. 2012; Dowker et al. 2016). This has led to a variety of studies which investigated the effects of targeting math anxiety/attitudes on subsequent math ability (Neale 1969; Lim and Chapman 2015). However, further research in this area investigated the direction of causality in the relation between math’s cognitive and affective factors and discovered that math emotions and math skills appear to develop reciprocally such that they influence each other across time (Lichtenfeld et al. 2022). This literature has also pointed to the importance of considering the level of balance between the factors in such a reciprocal relation. When it comes to interventions, if the factors are relatively balanced, one might anticipate that intervening on either factor would have similar long-term effects. However, if the factors are unbalanced, such that one is more predictive than the other, interventions should focus on the more predictive factor. In the case of math, evidence suggests that the reciprocal relation between math emotions and math skills is unbalanced, such that math skills are more predictive of later math anxiety over and above the autoregressive effects (Bellon et al. 2021; Gunderson et al. 2018).

Given the success in using longitudinal research to investigate the causality and reciprocity of cognitive and affective factors in the math domain, we advise that similar work should be conducted with respect to gender differences in spatial processing. Tentatively, our results indicate a bidirectional relation between gender differences in spatial ability and spatial anxiety, with the potential for the cognitive → affective relational direction being stronger than the reverse. To that end, our data lead us to the following prediction for future longitudinal and intervention work on gender differences in the spatial domain. The most successful interventions aimed at reducing gender differences in spatial processing will be those that focus on both cognitive and affective factors, as such interventions may benefit from reciprocal influences between cognitive and emotional responses. Of course, it is important to underscore the fact that these are merely predictions, not conclusions, as only future longitudinal and intervention work can provide a true test.

## 6. Conclusions

In this study we showed that (1) spatial anxiety mediates the relation between gender and spatial ability, and (2) spatial ability mediates the relation between gender and spatial anxiety. These results suggest a strong connection between cognitive and affective processing in the spatial domain and underscore the need to investigate both factors when investigating gender differences in spatial processing. This may be particularly important with respect to research on how gender differences in spatial processing may contribute to gender disparities in STEM representation. Furthermore, our results suggest a bidirectional relation between spatial ability and spatial anxiety, though we urge future longitudinal research in this area to better determine the directional reciprocity of this relation. Without such research, we strongly caution the development of interventions for addressing gender differences in spatial processing which focus primarily on either spatial anxiety or spatial ability.

## Figures and Tables

**Table 1:** Table lists study names and short names, sample sizes, number of female participants, and age distributions. References for any published work that has utilized data from each study’s sample of participants are also listed.

Dataset Source	Short Name	N	Female N	Mean Age
Geospatial Semester Study	GSS	105	58	16.61 (SD = 0.56)
(Cortes et al. 2022)				
Western University Study	Western	186	118	18.56 (SD = 0.42)
(Daker et al. 2022b)				
University of Ottawa Study	Ottawa	385	265	19.39 (SD = 4.7)
(Daker et al. 2022b)				
University of California, Los Angeles Study	UCLA	260	185	21.2 (SD = 3.6)
(Lyons et al. 2018)				
Georgetown University Online Study	Georgetown	170	133	20.21 (SD = 1.41)
(unpublished)				
Georgetown University Computer Science Study	GU-CS	151	71	20.57 (SD = 3.25)
(Daker et al. 2022a)				
Total		1257	830	19.42

**Table 2:** Table shows the average mediation effects for Hypothesis 1, that gender differences in spatial anxiety mediate gender differences in spatial ability, across all 6 datasets and across the 4 datasets with significant indirect effects (excluding GU-CS and GSS). ^†^ Denotes bootstrapped results (beta, standard error, and confidence intervals). * Excludes GU-CS and GSS datasets.

Hypothesis 1: Average Mediation Pathways			
	Number of Averaged Datasets	6	4 *
Unweighted Averages	A-Path ^†^	0.531	0.524
	B-Path ^†^	−0.222	−0.265
	Total Effect (C-Path)	−0.575	−0.640
	Direct Effect (C’-Path)	−0.458	−0.551
	Indirect Effect (A × B) ^†^	−0.116	−0.129
	Percent Mediation (Indirect/Total Effect %)	0.202 (20.2%)	0.202 (20.2%)
Weighted Averages	A-Path ^†^	0.531	0.424
	B-Path ^†^	−0.244	−0.217
	Total Effect (C-Path)	−0.571	−0.495
	Direct Effect (C’-Path)	−0.443	−0.382
	Indirect Effect (A × B) ^†^	−0.128	−0.113
	Percent Mediation (Indirect/Total Effect %)	0.224 (22.4%)	0.229 (22.9%)

**Table 3:** Table shows the average mediation effects for Hypothesis 2, that gender differences in spatial ability mediate gender differences in spatial anxiety, across all 6 datasets and across the 4 datasets with significant indirect effects (excluding GU-CS and GSS). ^†^ Denotes bootstrapped results (beta, standard error, and confidence intervals). * Excludes GU-CS and GSS datasets.

Hypothesis 2: Average Mediation Pathways			
	Number of Averaged Datasets	6	4 *
Unweighted Averages	A-Path ^†^	−0.575	−0.640
	B-Path ^†^	−0.209	−0.252
	Total Effect (C-Path)	0.531	0.524
	Direct Effect (C’-Path)	0.402	0.362
	Indirect Effect (A × B) ^†^	0.130	0.163
	Percent Mediation (Indirect/Total Effect %)	0.244 (24.4%)	0.311 (31.1%)
Weighted Averages	A-Path ^†^	−0.571	−0.495
	B-Path ^†^	−0.227	−0.203
	Total Effect (C-Path)	0.531	0.424
	Direct Effect (C’-Path)	0.394	0.297
	Indirect Effect (A × B) ^†^	0.138	0.127
	Percent Mediation (Indirect/Total Effect %)	0.259 (25.9%)	0.300 (30.0%)

## Data Availability

The raw data supporting the conclusions of this article will be made available by the authors on request.

## Notes

1 The reader may instead be motivated to consider, not the overall pattern of results, but the results of each test separately. In that case, the Dunn–Šidák (Šidák 1967) corrected threshold for an individual test result is *α* = 0.009. That said, it is important to consider how this perspective, which focuses on the strength of evidence from a single test in isolation instead of the combined weight of evidence across all six tests, is conceptually different from our approach looking at the overall pattern of results.

2 Studies specifically designed to investigate gender and sex use surveys which delineate between biological sex (designated as “male”, “female”, or “intersex”) and gender (designated as “man”, “woman”, or “non-binary”; Lips 2020). The surveys used in the present datasets do not differentiate between gender and biological sex. We utilize the terminology from these surveys throughout the manuscript.

## Appendix A

**Table A1:** Table shows the effect sizes (Cohen’s d) and corresponding confidence intervals for gender differences in MRT performance. Negative values indicate lower performance for women when compared to men. Average effect size includes GU-CS.

Gender Differences in MRT Performance							
	Cohen’s D	Confidence Interval		Mean MRT (std.)			
		Lower Bound	Upper Bound	Female		Male	
GSS	−0.907	−1.316	−0.499	−0.371	(1.08)	0.458	(0.653)
Western	−0.785	−1.096	−0.474	−0.269	(1.044)	0.467	(0.713)
Ottawa	−0.539	−0.758	−0.319	−0.163	(1.001)	0.36	(0.902)
UCLA	−0.636	−0.911	−0.361	−0.177	(0.95)	0.436	(0.992)
Georgetown	−0.481	−0.852	−0.111	−0.103	(0.993)	0.37	(0.949)
GU-CS	−0.049	−0.371	0.273	−0.026	(0.993)	0.023	(1.011)
Unweighted Average	−0.57			−0.18		0.35	
Weighted Average	−0.56						

**Table A2:** Table shows the effect sizes (Cohen’s d) and corresponding confidence intervals for gender differences in spatial anxiety. Positive values indicate higher spatial anxiety for women when compared to men. Average effect size includes GU-CS.

Gender Differences in Spatial Anxiety							
	Cohen’s D	Confidence Interval		Mean MRT (std.)			
		Lower Bound	Upper Bound	Female		Male	
GSS	0.892	0.484	1.3	0.366	(1.037)	−0.45	(0.741)
Western	0.641	0.334	0.948	0.224	(0.997)	−0.389	(0.886)
Ottawa	0.846	0.621	1.07	0.246	(0.956)	−0.543	(0.877)
UCLA	0.336	0.065	0.607	0.096	(1.019)	−0.237	(0.916)
Georgetown	0.839	0.461	1.217	0.173	(0.973)	−0.622	(0.845)
GU-CS	0.489	0.162	0.816	0.252	(0.978)	−0.224	(0.971)
Unweighted Average	0.67			0.23		−0.41	
Weighted Average	0.67						

## Appendix B

**Table A3:** Table shows the correlation matrices for all variables and covariates included in the mediation models for each of the 6 datasets.

**GSS**				
	**MRT**	**Gender**	**Spatial Anxiety**	**Trait Anxiety**
MRT	1			
Gender	r = −0.415	1		
	(p = 1.098 × 10^−5^)			
Spatial Anxiety	r = −0.307	r = 0.408	1	
	(p = 0.001)	(p = 1.505 × 10^−5^)		
Trait Anxiety	r = −0.111	r = 0.264	r = 0.434	1
	(p = 0.261)	(p = 0.007)	(p = 3.691 × 10^−6^)	
Age	r = −0.198	r = −0.045	r = 0.06	r = −0.031
	(p = 0.04)	(p = 0.647)	(p = 0.539)	(p = 0.752)
**Western**				
	**MRT**	**Gender**	**Spatial Anxiety**	**Trait Anxiety**
MRT	1			
Gender	r = −0.355	1		
	(p = 6.497 × 10^−7^)			
Spatial Anxiety	r = −0.306	r = 0.296	1	
	(p = 2.226 × 10^−5^)	(p = 3.988 × 10^−5^)		
Trait Anxiety	r = −0.088	r = 0.321	r = 0.313	1
	(p = 0.234)	(p = 7.922 × 10^−6^)	(p = 1.369 × 10^−5^)	
Age	r = −0.120	r = −0.204	r = −0.051	r = 0.021
	(p = 0.102)	(p = 0.005)	(p = 0.492)	(p = 0.779)
**Ottawa**				
	**MRT**	**Gender**	**Spatial Anxiety**	**Trait Anxiety**
MRT	1			
Gender	r = −0.243	1		
	(p = 1.443 × 10^−6^)			
Spatial Anxiety	r = −0.308	r = 0.366	1	
	(p = 6.51 × 10^−10^)	(p = 1.277 × 10^−13^)		
Trait Anxiety	r = −0.054	r = 0.258	r = 0.347	1
	(p = 0.288)	(p = 2.713 × 10^−7^)	(p = 2.405 × 10^−12^)	
Age	r = −0.06	r = −0.070	r = −0.054	r = 0.011
	(p = 0.242)	(p = 0.169)	(p = 0.296)	(p = 0.829)
**UCLA**				
	**MRT**	**Gender**	**Spatial Anxiety**	**Trait Anxiety**
MRT	1			
Gender	r = −0.278	1		
	(p = 5.339 × 10^−6^)			
Spatial Anxiety	r = −0.333	r = 0.151	1	
	(p = 3.913 × 10^−8^)	(p = 0.015)		
Trait Anxiety	r = 0.004	r = −0.028	r = 0.244	1
	(p = 0.953)	(p = 0.648)	(p = 7.116 × 10^−5^)	
Age	r = −0.002	r = 0.030	r = −0.0759	r = 0.0017
	(p = 0.969)	(p = 0.629)	(p = 0.222)	(p = 0.978)
**Georgetown**				
	**MRT**	**Gender**	**Spatial Anxiety**	**Trait Anxiety**
MRT	1			
Gender	r = −0.196	1		
	(p = 0.010)			
Spatial Anxiety	r = −0.291	r = 0.329	1	
	(p = 1.17 × 10^−4^)	(p = 1.195 × 10^−5^)		
Trait Anxiety	r = −0.195	r = 0.211	r = 0.324	1
	(p = 0.011)	(p = 0.006)	(p = 1.597 × 10^−5^)	
Age	r = 0.191	r = −0.2546	r = −0.088	r = −0.120
	(p = 0.013)	(p = 8.35 × 10^−4^)	(p = 0.2549)	(p = 0.119)
**GU-CS**				
	**MRT**	**Gender**	**Spatial Anxiety**	**Trait Anxiety**
MRT	1			
Gender	r = −0.025	1		
	(p = 0.763)			
Spatial Anxiety	r = -0.113	r = 0.238	1	
	(p = 0.169)	(p = 0.003)		
Trait Anxiety	r = −0.035	r = 0.141	r = 0.114	1
	(p = 0.667)	(p = 0.083)	(p = 0.165)	
Age	r = −0.032	r = −0.043	r = −0.014	r = 0.163
	(p = 0.694)	(p = 0.597)	(p = 0.865)	(p =0.046)

## Appendix C

Appendix C contains the results for Hypothesis 1: Does spatial anxiety explain (mediate) the relation between gender and spatial ability?

**Table A4:** Table shows the data from mediation models for Hypothesis 1. Mediation models constructed from these data points are depicted in Figure 3. ^†^ Denotes bootstrapped results (beta, standard error, and confidence intervals).

**GSS**				
	**beta**	**se**	**Confidence Interval**	
			**Lower Bound**	**Upper Bound**
A-Path ^†^	0.639	0.167	0.315	0.973
B-Path ^†^	−0.161	0.101	−0.365	0.029
Total Effect (C-Path)	−0.842	0.183	−1.205	−0.4791
Direct Effect (C’-Path)	−0.739	0.194	−1.123	−0.355
Indirect Effect (A × B) ^†^	−0.103	0.068	−0.248	0.019
Percent Mediation (Indirect/Total Effect %)	no med.			
**Western**				
	**beta**	**se**	**Confidence Interval**	
			**Lower Bound**	**Upper Bound**
A-Path ^†^	0.447	0.146	0.156	0.745
B-Path ^†^	−0.243	0.074	−0.383	−0.094
Total Effect (C-Path)	−0.855	0.152	−1.154	−0.556
Direct Effect (C’-Path)	−0.746	0.151	−1.044	−0.449
Indirect Effect (A × B) ^†^	−0.109	0.049	−0.218	−0.027
Percent Mediation (Indirect/Total Effect %)	12.70%			
**Ottawa**				
	**beta**	**se**	**Confidence Interval**	
			**Lower Bound**	**Upper Bound**
A-Path ^†^	0.638	0.1	0.447	0.8329
B-Path ^†^	−0.279	0.055	−0.39	−0.174
Total Effect (C-Path)	−0.549	0.111	−0.767	−0.33
Direct Effect (C’-Path)	−0.37	0.113	−0.592	−0.149
Indirect Effect (A × B) ^†^	−0.178	0.047	−0.277	−0.102
Percent Mediation (Indirect/Total Effect %)	32.50%			
**UCLA**				
	**beta**	**se**	**Confidence Interval**	
			**Lower Bound**	**Upper Bound**
A-Path ^†^	0.353	0.127	0.096	0.593
B-Path ^†^	−0.318	0.058	−0.434	−0.207
Total Effect (C-Path)	−0.613	0.132	−0.874	−0.352
Direct Effect (C’-Path)	−0.501	0.128	−0.752	−0.249
Indirect Effect (A × B) ^†^	−0.112	0.044	−0.205	−0.029
Percent Mediation (Indirect/Total Effect %)	18.30%			
**Georgetown**				
	**beta**	**se**	**Confidence Interval**	
			**Lower Bound**	**Upper Bound**
A-Path ^†^	0.657	0.16	0.339	0.985
B-Path ^†^	−0.219	0.077	−0.362	−0.060
Total Effect (C-Path)	−0.545	0.185	−0.909	−0.180
Direct Effect (C’-Path)	−0.401	0.189	−0.773	−0.029
Indirect Effect (A × B) ^†^	−0.144	0.063	−0.279	−0.032
Percent Mediation (Indirect/Total Effect %)	26.40%			
**GU-CS**				
	**beta**	**se**	**Confidence Interval**	
			**Lower Bound**	**Upper Bound**
A-Path ^†^	0.4507	0.161	0.117	0.755
B-Path ^†^	−0.112	0.077	−0.26	0.04
Total Effect (C-Path)	−0.044	0.167	−0.373	0.285
Direct Effect (C’-Path)	0.006	0.171	−0.331	0.343
Indirect Effect (A × B) ^†^	−0.05	0.042	−0.145	0.018
Percent Mediation (Indirect/Total Effect %)	no med.			

## Appendix D

Appendix D contains the results for Hypothesis 2: Does spatial ability explain (mediate) the relation between gender and spatial anxiety?

**Table A5:** Table shows the data from mediation models for Hypothesis 2. Mediation models constructed from these data points are depicted in Figure 4. ^†^ Denotes bootstrapped results (beta, standard error, and confidence intervals).

**GSS**				
	**beta**	**se**	**Confidence Interval**	
			**Lower Bound**	**Upper Bound**
A-Path ^†^	−0.842	0.178	−1.206	−0.500
B-Path ^†^	−0.145	0.087	−0.310	0.026
Total Effect (C-Path)	0.640	0.004	0.296	0.984
Direct Effect (C’-Path)	0.518	0.007	0.142	0.894
Indirect Effect (A × B) ^†^	0.122	0.078	−0.019	0.281
Percent Mediation (Indirect/Total Effect %)	no med.			
**Western**				
	**beta**	**se**	**Confidence Interval**	
			**Lower Bound**	**Upper Bound**
A-Path ^†^	−0.855	0.128	−1.109	−0.605
B-Path ^†^	−0.251	0.073	−0.386	−0.101
Total Effect (C-Path)	0.447	0.154	0.143	0.751
Direct Effect (C’-Path)	0.232	0.162	−0.088	0.553
Indirect Effect (A × B) ^†^	0.215	0.072	0.079	0.366
Percent Mediation (Indirect/Total Effect %)	48.00%			
**Ottawa**				
	**beta**	**se**	**Confidence Interval**	
			**Lower Bound**	**Upper Bound**
A-Path ^†^	−0.549	0.106	−0.754	−0.337
B-Path ^†^	−0.239	0.048	−0.336	−0.148
Total Effect (C-Path)	0.638	0.103	0.436	0.840
Direct Effect (C’-Path)	0.507	0.102	0.306	0.708
Indirect Effect (A × B) ^†^	0.131	0.036	0.067	0.208
Percent Mediation (Indirect/Total Effect %)	20.52%			
**UCLA**				
	**beta**	**se**	**Confidence Interval**	
			**Lower Bound**	**Upper Bound**
A-Path ^†^	−0.613	0.137	−0.874	−0.330
B-Path ^†^	−0.313	0.053	−0.416	−0.207
Total Effect (C-Path)	0.353	0.131	0.095	0.612
Direct Effect (C’-Path)	0.161	0.130	−0.095	0.417
Indirect Effect (A × B) ^†^	0.192	0.052	0.096	0.303
Percent Mediation (Indirect/Total Effect %)	54.36%			
**Georgetown**				
	**beta**	**se**	**Confidence Interval**	
			**Lower Bound**	**Upper Bound**
A-Path ^†^	−0.545	0.161	−0.869	−0.231
B-Path ^†^	−0.205	0.078	−0.358	−0.054
Total Effect (C-Path)	0.657	0.178	0.305	1.009
Direct Effect (C’-Path)	0.546	0.179	0.192	0.900
Indirect Effect (A × B) ^†^	0.114	0.056	0.023	0.241
Percent Mediation (Indirect/Total Effect %)	17.35%			
**GU-CS**				
	**beta**	**se**	**Confidence Interval**	
			**Lower Bound**	**Upper Bound**
A-Path ^†^	−0.044	0.158	−0.357	0.264
B-Path ^†^	−0.105	0.079	−0.277	0.033
Total Effect (C-Path)	0.451	0.161	0.132	0.770
Direct Effect (C’-Path)	0.446	0.161	0.128	0.764
Indirect Effect (A × B) ^†^	0.005	0.021	−0.034	0.056
Percent Mediation (Indirect/Total Effect %)	no med.			
